# Donor Treg expansion by liposomal α‐galactosylceramide modulates Tfh cells and prevents sclerodermatous chronic graft‐versus‐host disease

**DOI:** 10.1002/iid3.425

**Published:** 2021-05-04

**Authors:** Hiroyuki Sugiura, Ken‐ichi Matsuoka, Takuya Fukumi, Yuichi Sumii, Takumi Kondo, Shuntaro Ikegawa, Yusuke Meguri, Miki Iwamoto, Yasuhisa Sando, Makoto Nakamura, Tomohiro Toji, Yasuyuki Ishii, Yoshinobu Maeda

**Affiliations:** ^1^ Department of Hematology and Oncology Okayama University Graduate School of Medicine, Dentistry and Pharmaceutical Sciences Okayama Japan; ^2^ Department of Pathology Okayama University Hospital Okayama Japan; ^3^ REGiMMUNE Corporation Tokyo Japan; ^4^ Department of Immunological Diagnosis Juntendo University Graduate School of Medicine Tokyo Japan

**Keywords:** chronic graft‐versus‐host disease, hematopoietic stem cell transplantation, iNKT cells, regulatory T cells, Tfh cells, α‐galactosylceramide

## Abstract

**Background and Aim:**

Chronic graft‐versus‐host disease (cGVHD) is a major cause of nonrelapse morbidity and mortality following hematopoietic stem cell transplantation (HSCT). α‐Galactosylceramide (α‐GC) is a synthetic glycolipid that is recognized by the invariant T‐cell receptor of invariant natural killer T (iNKT) cells in a CD1d‐restricted manner. Stimulation of iNKT cells by α‐GC leads to the production of not only immune‐stimulatory cytokines but also immune‐regulatory cytokines followed by regulatory T‐cell (Treg) expansion in vivo.

**Methods:**

We investigated the effect of iNKT stimulation by liposomal α‐GC just after transplant on the subsequent immune reconstitution and the development of sclerodermatous cGVHD.

**Results:**

Our study showed that multiple administrations of liposomal α‐GC modulated both host‐ and donor‐derived iNKT cell homeostasis and induced an early expansion of donor Tregs. We also demonstrated that the immune modulation of the acute phase was followed by the decreased levels of CXCL13 in plasma and follicular helper T cells in lymph nodes, which inhibited germinal center formation, resulting in the efficient prevention of sclerodermatous cGVHD.

**Conclusions:**

These data demonstrated an important coordination of T‐ and B‐cell immunity in the pathogenesis of cGVHD and may provide a novel clinical strategy for the induction of immune tolerance after allogeneic HSCT.

## Graphical abstract

This study showed that multiple administrations of liposomal α‐Galactosylceramide (α‐GC) modulated both host‐ and donor‐derived invariant natural killer T (iNKT) cell homeostasis and induced an early expansion of donor Tregs. This was followed by the decreased levels of CXCL13 in plasma and follicular helper T cells in lymph nodes, which inhibited germinal center formation, resulting in the efficient prevention of sclerodermatous chronic graft‐versus‐host disease (GVHD)."

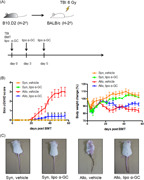

**Abbreviations**
BM: bone marrow
BMT: bone marrow transplantation
GVHD: graft‐versus‐host disease
cGVHD: chronic GVHD
Syn: syngeneic
Allo: allogeneic
α‐GC: α‐galactosylceramide
lipo α‐GC: liposomal α‐GC
iNKT cell: invariant NKT cell
Treg: regulatory T cell
Tfh: follicular helper T cell
GCB cell: germinal center B cell
HSCT: hematopoietic stem cell transplantation

## 1 INTRODUCTION

Chronic graft‐versus‐host disease (cGVHD) is a major cause of nonrelapse morbidity and mortality following hematopoietic stem cell transplantation (HSCT),^1^ while effective prophylaxis and therapies for cGVHD are still lacking.^2^ CD4^+^CD25^+^Foxp3^+^ regulatory T cells (Tregs) play an indispensable role in the maintenance of tolerance after allogeneic HSCT.^3^ It has been reported that adoptive transfer of donor‐type Tregs could prevent the onset of acute GVHD in murine bone marrow transplantation (BMT) models and human clinical trials,^4^ and the administration of low‐dose interleukin‐2 (IL‐2) to patients with active cGVHD increased the levels of peripheral Tregs and ameliorated the clinical symptoms.^5^, ^6^ These results suggest that in vivo modulation of Tregs is a promising strategy to control aggressive GVHD after allogeneic HSCT.

α‐Galactosylceramide (α‐GC) is a synthetic glycolipid, which is recognized by the invariant T‐cell receptor of invariant natural killer T (iNKT) cells in a CD1d‐restricted manner.^7^ Stimulation of iNKT cells by α‐GC leads to the production of not only immune‐stimulatory cytokines but also immune regulatory cytokines such as IL‐4 and IL‐10, followed by Treg expansion in vivo.^8^ Previous studies in mice have shown that the administration of an aqueous α‐GC stimulated host‐type iNKT cells and ameliorated acute GVHD by expanding donor Tregs via production of IL‐4.^9^, ^10^ Subsequently, a liposomal α‐GC (lipo α‐GC) was developed and reported to be tolerogenic and safe in vivo^11^; furthermore, its acute GVHD‐preventing effect was proven in a murine model.^12^ Based on these findings, a phase 2 clinical trial was performed, which demonstrated that the administration of lipo α‐GC immediately after HSCT contributed to the prevention of acute GVHD, and the effects were dependent on Treg expansion.^13^ Moreover, the administration of lipo α‐GC prevented and ameliorated cGVHD in a lung cGVHD mouse model.^14^

The significance of B‐cell immunity in cGVHD has been increasingly recognized. Thus, it has been reported that an abnormality in B‐cell homeostasis was related to the development of cGVHD in humans.^15^ In addition, it has been observed that T follicular helper (Tfh) cells played a role in cGVHD patients. A recent analysis of clinical samples from post‐HSCT patients demonstrated that Tfh cells were significantly reduced in the peripheral blood and the plasma concentration of CXCL13, a CXCR5 ligand, was elevated in patients with active cGVHD, suggesting that migration of Tfh cells from peripheral blood to lymphoid organs is one of the important processes in cGVHD.^16^ Recent studies in mice have shown that the excessive reaction between Tfh cells and germinal center B (GCB) cells resulted in the deposition of the IgG alloantibody, leading to tissue damage in cGVHD target organs.^17^, ^18^ Based on these findings, inhibitors of Bruton's tyrosine kinase and spleen tyrosine kinase, which are downstream signaling molecules of the B‐cell receptor, have been studied as treatments for steroid‐resistant cGVHD.^19^, ^20^, ^21^

It is known that patients with active cGVHD have a lower frequency of Tregs than do patients without cGVHD.^22^ Recently, an immunological crosstalk between Tregs and B cells has been reported in various fields. Tregs and T follicular regulatory cells were shown to suppress Tfh‐cell activation and germinal center formation, which could control humoral immunity in mice after vaccination.^23^ Adoptive transfer of donor Tregs allowed their infiltration into the germinal center and modulated B‐cell differentiation, resulting in amelioration of murine cGVHD in a CXCR5‐dependent manner.^24^

Here, we hypothesized that donor Treg expansion by lipo α‐GC modulates B‐cell immunity after HSCT and prevent the onset of cGVHD. To explore this hypothesis, we investigated the clinical effects and immune mechanisms of lipo α‐GC in a murine sclerodermatous cGVHD model.

## 2 MATERIALS AND METHODS

### 2.1 Mice

Female BALB/c (H‐2d) and B10.D2 (H‐2d) mice were purchased from Japan SLC. The mice were 10 weeks of age at the time of BMT. All mice were housed under specific pathogen‐free conditions and treated in strict compliance. All animal experiments were performed according to the regulations of the Animal Care and Use Committee, Okayama University Advanced Science Research Center.

### 2.2 Mouse models and assessment of skin cGVHD

To generate an allogeneic mouse model, BALB/c mice received 6 Gy of total‐body irradiation (TBI), which consisted of two doses administered at a 6‐h interval to minimize gastrointestinal toxicity. The irradiated host mice were intravenously injected with 10 × 10^6^ bulk spleen cells and 8 × 10^6^ bone marrow (BM) cells from B10.D2 mice. In a syngeneic mouse model, BALB/c mice received the same dose of TBI and were intravenously injected with the same doses of spleen and BM cells from BALB/c mice. Mice were monitored twice a week and scored for skin manifestations of GVHD. The following scoring system was used: healthy appearance, 0; skin lesions with an alopecia area < 1 cm^2^, 1; skin lesions with an alopecia area of 1–2 cm^2^, 2; and skin lesions with an alopecia area > 2 cm^2^, 3. In addition, animals were assigned 0.3 points each for skin disease (lesions or scaling) on the ears, tail, and paws. The minimum score was 0, and the maximum score was 3.9.^25^

### 2.3 Administration of lipo α‐GC

RGI‐2001 (lipo α‐GC) was provided by REGiMMUNE. We administered either lipo α‐GC (at a dose of 1 μg/kg) or a saline vehicle by tail‐vein injection on Days 0, 3, and 5 after BMT.

### 2.4 Administration of anti‐IL‐4 antibody

Anti‐IL4 antibody (Ultra‐LEAF^TM^ Purified antimouse IL‐4, Biolegend) was administered intraperitoneally on Days 0, 3, and 5 after BMT. Administration doses of the antibody were 2 mg on Day 0, and 1 mg on Days 3 and 5, respectively.

### 2.5 Depletion of CD25^+^ cells from the spleen cell population

Depletion of CD25^+^ cells was performed using a PE‐conjugated anti‐CD25 monoclonal antibody and microbeads (Miltenyi Biotec). Whole‐spleen cells harvested from donor mice were labeled with PE‐CD25 and anti‐PE microbeads, and CD25^−^ cells were negatively purified by magnetic separation using an AutoMACS system (Miltenyi Biotec). The purity of the CD25^−^CD4^+^ T‐cell fractions was above 95%.

### 2.6 Flow cytometry

Single‐cell suspensions were first incubated with the following directly conjugated monoclonal antibodies (obtained from eBioscience, unless stated otherwise) for 20 min at 4°C: eFluor450‐conjugated anti‐B220 (RA3‐6B2), anti‐CD4 (GK1.5), and anti‐TCR‐β (H57‐597); FITC‐conjugated anti‐GL7 (GL7; BioLegend) and anti‐Ly9.1 (30C7; BD Bioscience); phycoerythrin (PE)‐conjugated anti‐PD‐1 (RMP1‐30) and anti‐α‐GC‐loaded CD1d tetramer (Proimmune); PE‐Cy7‐conjugated anti‐CD25 (PC61.5); allophycocyanin (APC)‐conjugated anti‐Fas (SA367H8, BioLegend); and APC‐eFluor780‐conjugated anti‐CXCR5 (L138D7, BioLegend) and anti‐CD19 (eBio1D3). For intracellular staining, cells were processed using a Foxp3 staining buffer set (eBioscience) and then incubated with APC‐conjugated anti‐Foxp3 (FJK‐16S) for 30 min at 4°C. Samples were analyzed using a MAQSQuant flow cytometer (Miltenyi Biotec), and data were analyzed using the FlowJo software version 10 (TreeStar). flow cytometry gating strategy for specific lymphocyte subsets is shown in Figures S1 and S2.

### 2.7 Enzyme‐linked immunosorbent assay

Mouse plasma was collected on Day 56 following HSCT, and plasma samples were assayed for CXCL13 via sandwich enzyme‐linked immunosorbent assay using the Quantikine mouse CXCL13/BLC/BCA‐1 immunoassay (R&D Systems). Samples were diluted fivefold with the calibrator diluent and tested along with standards and calibrator controls, following the manufacturer's instructions.

### 2.8 Tissue histopathology

A shaved skin from the interscapular region (~2 cm^2^) of recipients was fixed in 10% formalin, embedded in paraffin, sectioned, mounted on slides, and stained with hematoxylin and eosin. Slides were scored by a pathologist (T. T.) on the basis of dermal fibrosis, fat loss, inflammation, epidermal interface changes, and follicular dropout (0–2 for each category, with the maximum score of 10).^26^

### 2.9 Statistical analysis

Group comparisons of skin cGVHD scores and skin pathological scores were performed using the Mann–Whitney *U*‐test or Kruskal–Wallis test. Cell populations and cytokine levels were analyzed using an unpaired two‐tailed Student's *t *test or one‐way analysis of variance with Tukey's post hoc test using Prism 7 (GraphPad Software). Spearman's rank correlation analysis was used to examine the correlation between the percentage of Tfh cells and the CXCL13 level, skin cGVHD score, and skin pathological score, respectively, and the coefficients were calculated using EZR (Saitama Medical Center, Jichi Medical University, Saitama, Japan), a graphical user interface for R (R Foundation for Statistical Computing, Vienna, Austria).^27^

## 3 RESULTS

### 3.1 Administration of lipo α‐GC improved the skin cGVHD score and body weight

**Figure 1:**
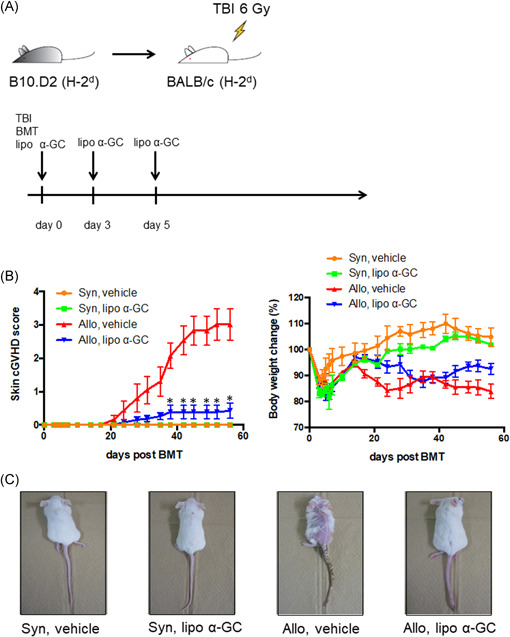
Lipo α‐GC prevents cGVHD in a sclerodermatous cGVHD mouse model. (A) Experimental scheme. BALB/c recipient mice received 6 Gy of TBI; then, 8 × 10^6^ BM cells and 10 × 10^6^ spleen cells were administered from B10.D2 donor mice. Lipo α‐GC was administered at a dose of 1 μg/kg via tail vein injection on Days 0, 3, and 5 after BMT. (B) Time courses of skin cGVHD score and percent body weight changes from baseline after BMT in syngeneic/vehicle (*n* = 3), syngeneic/lipo α‐GC (*n* = 3), allogeneic/vehicle (*n* = 8), and allogeneic/lipo α‐GC (*n* = 8) groups. Representative data from one of three independently performed experiments are expressed as the means ± *SEM. p*‐values were determined by the Kruskal–Wallis test; *p < .05. (C) Representative photographs of mice from syngeneic/vehicle (*n* = 3), syngeneic/lipo α‐GC (*n* = 3), allogeneic/vehicle (*n* = 8), and allogeneic/lipo α‐GC (*n* = 8) groups on Day 56 after BMT from one of three independently performed experiments. BMT, bone marrow transplantation; BM, bone marrow; cGVHD, chronic graft‐versus‐host disease; α‐GC, α‐Galactosylceramide

**Figure 2:**
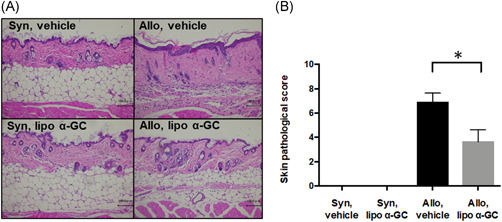
Lipo α‐GC reduces pathological scores in a sclerodermatous cGVHD mouse model. (A) Representative photomicrographs of hematoxylin‐ and eosin‐stained skin sections from syngeneic/vehicle, syngeneic/lipo α‐GC, allogeneic/vehicle, and allogeneic/lipo α‐GC mice on Day 56 after BMT. Scale bar = 100 μm. ×20. (B) Pathological scores of skin cGVHD in syngeneic/vehicle (*n* = 2, one experiment), syngeneic/lipo α‐GC (*n* = 2, one experiment), allogeneic/vehicle (*n* = 8, data combined from two independently performed experiments with four mice per experiments), and allogeneic/lipo α‐GC (*n* = 8, data combined from two independently performed experiments with four mice per experiments) groups on Day 56. Data are expressed as the means ± *SEM. p‐*values were determined by the Mann–Whitney *U*‐test (allogeneic/vehicle vs. allogeneic/lipo α‐GC); *p < .05. BMT, bone marrow transplantation; cGVHD, chronic graft‐versus‐host disease; α‐GC, α‐Galactosylceramide

To investigate whether the administration of lipo α‐GC prevents sclerodermatous cGVHD, allogeneic and syngeneic BMT mice were treated with lipo α‐GC or the vehicle and monitored for the cGVHD clinical score and body weight (Figure 1A). The allogeneic host mice treated with lipo α‐GC (allo lipo α‐GC group) showed significantly lower skin cGVHD scores than did the vehicle‐treated control allogeneic host mice (allo vehicle group). The body weight tended to be higher in the allo lipo α‐GC group than in the allo vehicle group, although there was no significant difference (Figure 1B). The appearance of the mouse skin on Day 56 was consistent with the clinical score (Figure 1C). The pathological score of skin cGVHD in the lipo α‐GC‐treated allogeneic BMT mice was significantly lower than that in the vehicle‐treated allogeneic mice (Figure 2A,B). The results collectively suggested that lipo α‐GC treatment could protect from the development of sclerodermatous cGVHD in the allogeneic BMT mouse model.

### 3.2 Donor iNKT cells increased, host iNKT cells decreased, and donor tregs expanded in an early phase after BMT

**Figure 3:**
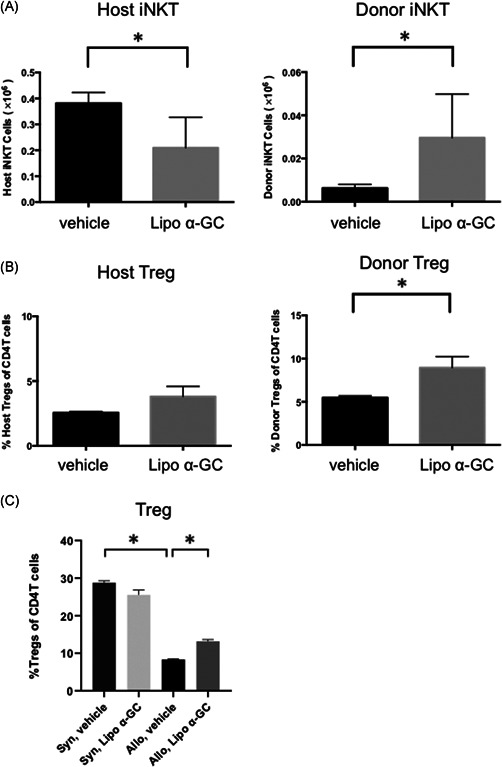
Lipo α‐GC increases donor iNKT cells, decreases host iNKT cells, and expands donor Tregs in an early phase after BMT. Tregs were defined as CD4^+^FoxP3^+^CD25^+^ cells and iNKT cells were defined as CD19^−^TCR‐β^+^CD1d tetramer^+^ cells. Chimerism was evaluated in the host (Ly9.1^+^) or donor (Ly9.1^−^). (A) The number of donor iNKT cells and host iNKT cells in spleen cells after BMT in vehicle (*n* = 5, one experiment) and lipo α‐GC (*n* = 5, one experiment) mice on Day 7. Data are expressed as means ± *SEM. p‐*values were determined using an unpaired, two‐tailed Student's *t *test. *p < .05. (B) Percentages of donor Tregs, and host Tregs among CD4^+^ T cells in spleen cells in vehicle (*n* = 5, one experiment) and lipo α‐GC (*n* = 5, one experiment) mice on Day 7. Data are expressed as means ± *SEM. p‐*values were determined using an unpaired, two‐tailed Student's *t *test. *p < .05. (C) Percentages of total Tregs among CD4^+^ T cells in spleen cells in syngeneic/vehicle (*n* = 3, one experiment), syngeneic/lipo α‐GC (*n* = 3, one experiment), allogeneic/vehicle (*n* = 5, one experiment) and allogeneic/lipo α‐GC (*n* = 5, one experiment) mice on Day 7. Data are expressed as means ± *SEM. P‐*values were determined using an unpaired, two‐tailed Student's *t *test. *p < .05. BMT, bone marrow transplantation; iNKT, invariant natural killer T; α‐GC, α‐Galactosylceramide

To clarify the mechanism of the preventive effect of lipo α‐GC against cGVHD in an early phase after BMT, we examined the dynamics of iNKT cells and Tregs by flow cytometric analysis of spleen cells on Day 7 (Figure 3). The analysis revealed that the number of donor iNKT cells in the lipo α‐GC‐treated mice was significantly higher on Day 7 than that in the vehicle‐treated mice, while that of host iNKT cells was inversely suppressed on Day 7 in the spleen (Figure 3A). We confirmed the kinetics of iNKT cells in lymph nodes was similar to that in the spleen (Figure S8). The proportion of donor but not host Tregs in the lipo α‐GC‐treated mice was significantly higher on Day 7 than that in the vehicle‐treated mice (Figure 3B). Tregs in the syngeneic cohorts, where the inflammation is relatively mild, are basically higher than Tregs in the allogeneic cohorts (Figure 3C). Addition of lipo α‐GC increased Treg in allogeneic cohorts, but the effect was not observed in syngeneic cohorts. These data suggested that multiple lipo α‐GC administrations modulate the homeostasis of both host‐ and donor‐derived iNKT cells, leading to preferential expansion of donor Tregs after allogeneic transplantation.

### 3.3 Preventive effect of lipo α‐GC against cGVHD was canceled by anti‐IL‐4 antibody

To consider whether the preventive effect of lipo α‐GC against cGVHD is in an IL‐4 dependent manner, we performed the additional injection of anti‐IL‐4 antibody to sclerodermatous cGVHD mouse model treated by lipo α‐GC (Figure S7). The additional injection of anti‐IL‐4 antibody to lipo α‐GC treated mice on Days 0, 3, and 5 resulted in the development of severe sclerodermatous GVHD. The data suggest that the tolerogenic activity of iNKT cells given by lipo α‐GC administration was at least partially dependent on the production of IL‐4.

### 3.4 Depletion of CD25‐positive cells canceled the expansion of donor Tregs on Day 7 and the preventive effect of lipo α‐GC against cGVHD

**Figure 4:**
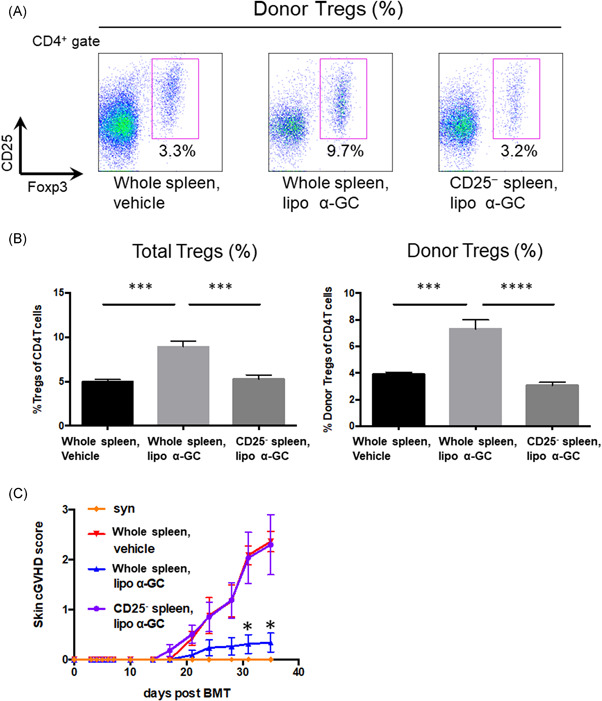
CD25 depletion inhibits donor Treg expansion and exacerbates cGVHD scores with lipo α‐GC treatment. (A) Representative flow cytometry images of donor Tregs (CD4^+^Ly9.1^−^FoxP3^+^CD25^+^) in whole spleen/vehicle, whole spleen/lipo α‐GC, and CD25‐depleted spleen/lipo α‐GC mice on Day 7. (B) Tregs and donor Tregs in whole spleen/vehicle (*n* = 5, one experiment), whole spleen/lipo α‐GC (*n* = 5, one experiment), and CD25‐depleted spleen/lipo α‐GC (*n* = 5, one experiment) groups. Data are expressed as means ± *SEM. P*‐values were determined by one‐way analysis of variance with Tukey's post hoc test. ***p < .001; ****p < .0001. (C) Skin cGVHD scores in syngeneic (*n* = 3, one experiment), whole allogeneic spleen/vehicle (*n* = 11, data combined from two independently performed experiments with five to six mice per experiments), whole allogeneic spleen/lipo α‐GC (*n* = 11, data combined from two independently performed experiments with five to six mice per experiments), and allogeneic CD25‐depleted spleen/lipo α‐GC (*n* = 11, data combined from two independently performed experiments with five to six mice per experiments) groups. Data are expressed as means ± *SEM. p*‐values were determined by the Kruskal–Wallis test. *p < .05. cGVHD,chronic graft‐versus‐host disease; α‐GC, α‐Galactosylceramide

To clarify the role of donor Tregs in the preventive effect of lipo α‐GC against cGVHD, we depleted CD25‐positive cells from the population of donor spleen cells. As shown in Figure 4A,B the proportion of donor Tregs in the mice that received CD25‐depleted spleen cells was significantly lower than that in the mice that received whole spleen cells, regardless of the lipo α‐GC treatment. In parallel, the protection against sclerodermatous cGVHD by lipo α‐GC treatment was canceled by the transfer of CD25‐depleted spleen cells (Figure 4C). The results indicated that CD25‐positive cells in the population of donor spleen cells were indispensable for the protection against sclerodermatous cGVHD by lipo α‐GC treatment.

### 3.5 Follicular helper T cells and GCB cells decreased in a late phase after BMT

**Figure 5:**
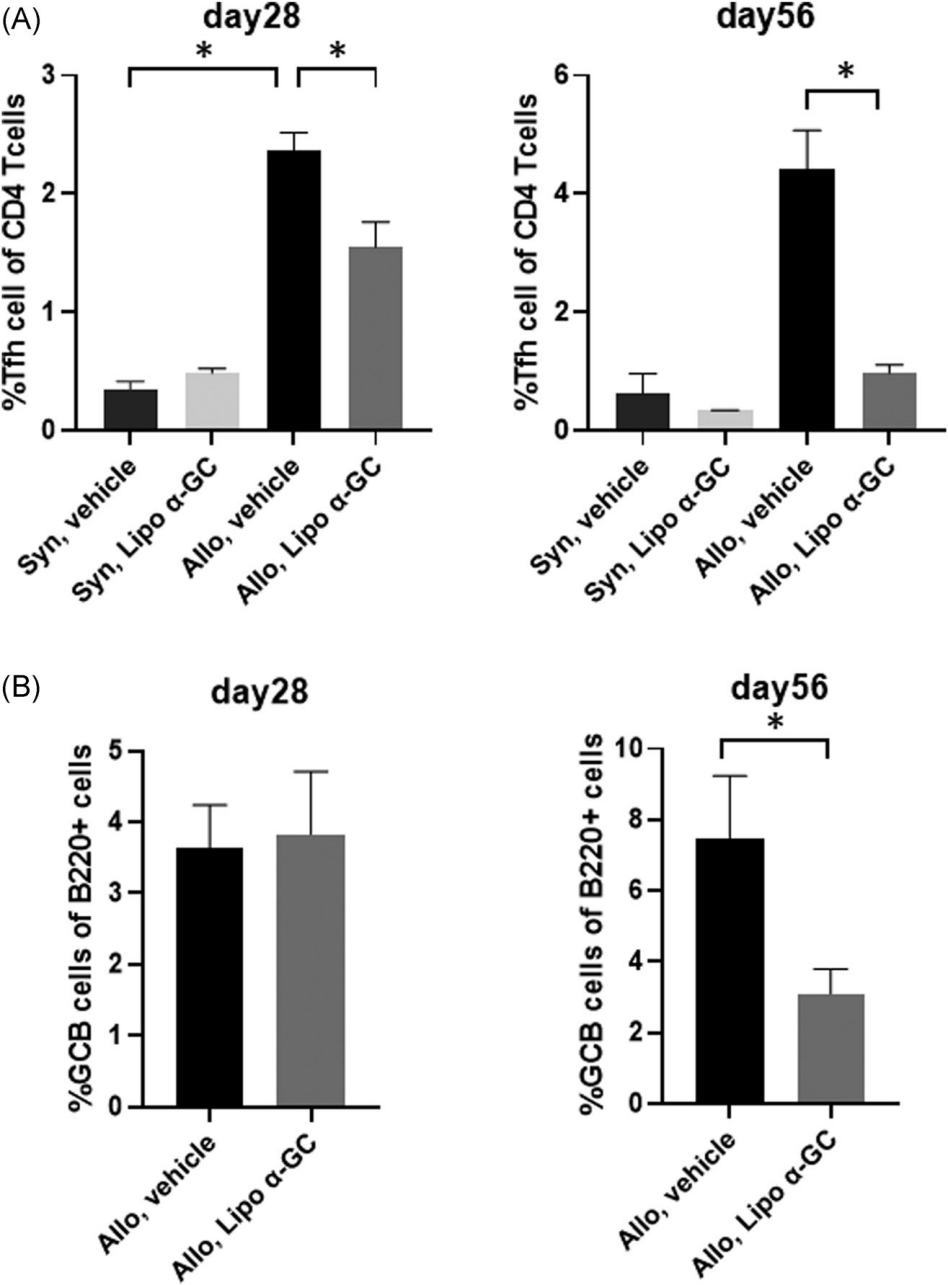
Lipo α‐GC inhibits Tfh and GCB cells in a late phase after BMT. Tfh cells were defined as CD4^+^CXCR5^+^PD‐1^+^ cells and GCB cells were defined as B220^+^GL7 ^+^Fas^+^ cells. (A) Proportions of Tfh cells in MLNs of syngeneic/vehicle (*n* = 3), syngeneic/lipo α‐GC (*n* = 3), allogeneic/vehicle (*n* = 8) and allogeneic/lipo α‐GC (*n* = 8) groups on Days 28 and 56. Data were combined from two independently performed experiments with four mice per experiments and are expressed as the means ± *SEM. P‐*values were determined using an unpaired, two‐tailed Student's *t *test. *p < .05. (B) Proportions of GCB cells in MLNs of vehicle (*n* = 8), and lipo α‐GC (*n* = 8) groups on Days 28 and 56. Data were combined from two independently performed experiments with four mice per experiments and are expressed as the means ± *SEM. P‐*values were determined using an unpaired, two‐tailed Student's *t *test. *p < .05. GCB, germinal center B cell; MLN, mesenteric lymph nodes; Tfh, follicular helper T cell; α‐GC, α‐Galactosylceramide

To clarify the mechanism of the preventive effect of lipo α‐GC against cGVHD in a late phase after BMT, we analyzed the proportions of Tfh and GCB cells in mesenteric lymph nodes (MLNs) by flow cytometry on Days 28 and 56 (Figure 5A,B). The results showed that Tfh in the syngeneic cohorts were in the lower levels than Tfh in the allogeneic cohorts. Of note, the lipo α‐GC treatment significantly decreased Tfh in allogeneic cohorts on Days 28 and 56. As well, the lipo α‐GC treatment significantly reduced GCB cells on Day 56. These results suggested that the tolerogenic effect of lipo α‐GC in the late phase after BMT was associated with the reduction of both Tfh and GCB cells in MLNs.

### 3.6 Plasma CXCL13 tended to decrease in the allo lipo α‐GC group and was correlated with tfh cells in MLNs

**Figure 6:**
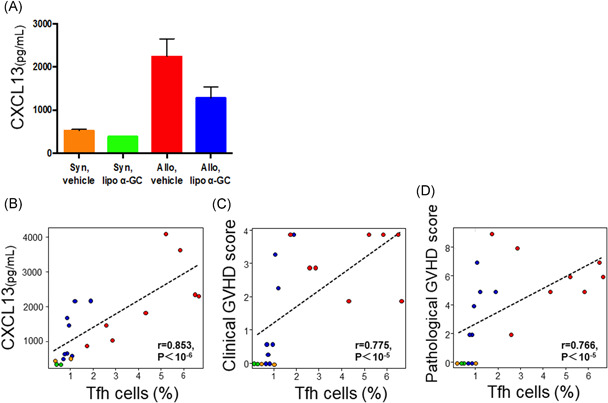
CXCL13 concentration increases in the allo lipo α‐GC group and is correlated with the proportion of Tfh cells. (A) Concentrations of CXCL13 in the plasma of syngeneic/vehicle (*n* = 2, one experiment), syngeneic/lipo α‐GC (*n* = 2, one experiment), allogeneic/vehicle (*n* = 8, data combined from two independently performed experiments with four mice per experiments), and allogeneic/lipo α‐GC (*n* = 8, data combined from two independently performed experiments with four mice per experiments) mice on Day 56. Data are expressed as means ± *SEM*. (B–D) Each orange, green, red, and blue marker indicates the result from syngeneic/vehicle, syngeneic/lipo α‐GC, allogeneic/vehicle, and allogeneic/lipo α‐GC, respectively. (B) Spearman's rank correlation between the percentage of Tfh cells in MLNs and the plasma concentration of CXCL13 on Day 56 (*r* = .853, *p* < 10^−6^). (C) Spearman's rank correlation between the percentage of Tfh cells in MLNs and the skin cGVHD score on Day 56 (*r* = .775, *p* < 10^−5^). (D) Spearman's rank correlation between the percentage of Tfh cells in MLNs and the skin pathological score on Day 56 (*r* = .766, *p* < 10^−5^). cGVHD, chronic graft‐versus‐host disease; MLN, mesenteric lymph nodes; Tfh, follicular helper T cell; α‐GC, α‐Galactosylceramide

Next, we evaluated whether the migration of Tfh cells into MLNs after BMT could be inhibited by lipo α‐GC treatment, by measuring the plasma level of CXCL13, a ligand for CXCR5, which is one of representative markers of Tfh cells. The plasma concentration of CXCL13 in the lipo α‐GC‐treated allogeneic mice tended to be lower than that in the vehicle‐treated allogeneic mice on Day 56 (Figure 6A). Notably, the concentration of CXCL13 in the plasma was correlated with the proportion of Tfh cells in the population of CD4^+^ T cells in MLNs (Figure 6B). Furthermore, the proportion of Tfh cells in MLNs on Day 56 was correlated with the skin cGVHD score and skin pathological score (Figure 6C,D). Taken together, these results suggested that the prevention of cGVHD symptoms by lipo α‐GC might be caused by the reduction of the CXCL13 in the periphery, which may inhibit the migration of Tfh cells into MLNs.

## 4 DISCUSSION

In this study, we demonstrated that multiple lipo α‐GC administrations were effective for the prevention of disease symptoms, such as the collapse of the skin and subcutaneous tissue, in a mouse model of sclerodermatous cGVHD. We also found that the number of host iNKT cells in lipo α‐GC‐treated mice was less than that in control mice on Day 7, while the number of donor iNKT cells in lipo α‐GC‐treated mice was significantly higher than that in control mice on Day 7. Our previous studies using BMT mouse models suggested that host iNKT cells were indispensable for the induction of immune tolerance after a single injection of lipo α‐GC.^12^, ^28^, ^29^ Other studies using murine acute BMT models also showed that host‐derived iNKT cells played a critical role in aqueous α‐GC‐induced immune tolerance, mediated by the production of IL‐4 and IL‐10.^9^, ^10^ Our study showed the preventive effect of sclerodermatous GVHD by lipo α‐GC was canceled by anti‐IL‐4 antibody, suggesting that the tolerogenic activity of iNKT cells is in an IL‐4‐dependent manner. Consistent with our results, a previous study reported that the direct administration of IL‐4 resulted in the reduction of Tfh.^30^ This may suggest a potential role of type‐2 cytokine in the prevention of cGVHD.

One might argue that the host iNKT cells in our model were not involved in the preventive effect because the number of cells decreased in the spleen. However, we consider that the disappearance of host iNKT cells from the periphery was caused by their rapid migration, which was activated by multiple lipo α‐GC administrations to the GVHD target organ. In fact, we tried to check iNKT cells in the skin as the cGVHD target organ. To detect iNKT cells in the skin, we dissolved the skin with Multi Tissue Dissolution Kit 1 (Miltenyi Biotic) and stained cells with CD45, CD19, TCR‐β, and CD1d tetramer. Unfortunately, however, it was very difficult to identify iNKT cells from the skin of recipients by flow cytometry (data not shown). This suggests that there are very few NKT cells in the skin on Day 7 in recipient mice that have undergone TBI. Accurate evaluation of this small subset of cells on GVHD target organs immediately after transplantation was considered to require the development of new methods. The evaluation of skin‐resident iNKT cells will be needed to further develop NKT‐based therapy in the future.

Simultaneously, the results also suggest that multiple lipo α‐GC administrations, rather than a single administration, may accelerate the early emergence of donor iNKT cells, followed by remarkable preventive effects. Indeed, recent adoptive transfer studies have reported that the administration of purified donor‐type iNKT cells, or even third‐party iNKT cells, potently suppressed GVHD.^31^, ^32^ Furthermore, similar results were observed in a systemic lupus erythematosus model, in addition to GVHD models.^33^, ^34^ In this study, we found that the proportion of donor Tregs among CD4^+^ T cells was much higher than that of host Tregs, and donor Tregs were indispensable for the prevention of cGVHD symptoms. Although this preferential expansion of donor Tregs after the treatment is beneficial for the prevention of GVHD, the mechanism still remains unclear. However, we hypothesize that multiple lipo α‐GC administrations modulate the homeostasis of both host‐ and donor‐derived iNKT cells, leading to preferential expansion of donor Tregs.

Various studies investigated the mechanisms of how the activation of NKT cells result in Treg recruitment and function and suggested the importance of IL‐4 and IL‐10 in the process.^9^, ^10^, ^35^ A recent study demonstrated that activated NKT cells produce IL‐2 which directly acts on Treg homeostasis.^26^ Further study will warrant the mechanism more precisely and develop NKT‐based therapy for immune tolerance.

Recent studies have suggested the importance of B‐cell immunity in the development of cGVHD. Analysis of clinical samples from patients with cGVHD demonstrated that the number of Tfh cells was significantly reduced in the peripheral blood and the plasma concentration of CXCL13, a CXCR5 ligand, increased in patients with active cGVHD, suggesting that the migration of Tfh cells from peripheral blood to lymphoid organs is one of the important processes in the development of cGVHD.^16^ Studies in mice have shown that the germinal center reaction between Tfh and GCB cells plays a critical role in cGVHD pathogenesis, and inhibition of this reaction significantly reduces cGHVD.^17^, ^18^ In our study, Tfh and GCB cells significantly decreased in MLNs of recipients treated with lipo α‐GC, in line with clinical amelioration of sclerodermatous cGVHD. In addition, the concentration of CXCL13 in peripheral blood of lipo α‐GC‐treated allogeneic recipients was substantially lower than that in vehicle‐treated allogeneic recipients and was correlated with the proportion of Tfh cells among CD4^+^ T cell. Furthermore, the proportion of Tfh cells was significantly correlated with the skin cGVHD score and skin pathological score. Based on these results, it was suggested that the preventive effects of lipo α‐GC against cGVHD might be related to the inhibition of Tfh and GCB cells. It appears that the decreased level of CXCL13 after lipo α‐GC treatment is also an important factor in this process of cGVHD prevention, needed to avoid the migration of Tfh cells to lymphoid organs.

It is known that patients with active cGVHD have a lower frequency of Tregs than do those without cGVHD.^22^ Recent studies have revealed an immunological crosstalk between Tregs and B cells, which is important for the germinal center formation in lymph nodes and B‐cell lymphopoiesis in the BM. Tregs and T follicular regulatory cells suppress Tfh‐cell activation and the germinal center formation, which can control humoral immunity in mice after vaccination.^23^ Adoptively transferred donor‐derived Tregs infiltrated into the germinal center and modulated B‐cell differentiation, resulting in amelioration of cGVHD in a CXCR5‐dependent manner in mice.^24^ In murine BMT models, B‐cell lymphopoiesis is impaired in host Treg‐depleted mice but is rescued by adoptive transfer of affected hematopoietic stem cells or BM cells into Treg‐competent recipients.^36^ On the other hand, infusion of splenic T cells, in which donor Tregs were expanded by an anti‐DR3 antibody, improved B‐cell lymphopoiesis in a mouse cGVHD model.^37^ These studies suggest that both host and donor Tregs are essential for B‐cell lymphopoiesis in HSCT. Compared with those of previous studies that used an adoptive Treg transfer system, our results suggest that in vivo expansion of donor Tregs by lipo α‐GC may regulate the germinal center reaction and restore B‐cell homeostasis in a cGVHD model. In fact, the preventive effect of lipo α‐GC against cGVHD was significantly canceled by the depletion of CD25‐positive cells from the population of donor spleen cells. We propose that the expansion of Tregs in a donor inoculum in a very acute phase may play a critical role in the regulation of pathogenic B‐cell immunity, contributing to the development of cGVHD.

Also, previous studies already showed that IL‐10 has a role in NKT‐mediated tolerance.^38^ In addition, a recent study has shown that IL‐10 produced by B cells is important to suppress GVHD.^39^ We have previously reported that lipo α‐GC is preferentially taken up by B cells and further promotes IL‐10 production from B cells.^11^ These results suggest that B‐cell/IL‐10‐axis has a critical role in the suppression of GVHD by lipo α‐GC. Further experiments using an IL‐10 blocking system may elucidate the effect and the mechanism of lipo α‐GC on cGVHD.

The relative proportion of iNKT to CD4+ T cells, Treg cells, Tfh cells, and GCB cells and Treg to CD4+ T cells, Tfh cells, GCB cells, and iNKT cells in the spleen on Days 7, 28, and 56 is shown in Figures S3 and S4. The relative proportion of iNKT to Treg in the spleen on Day 7 decreased in the lipo α‐GC group significantly. We assumed it might reflect increasing Treg and decreased host iNKT in the lipo α‐GC group. The relative proportion of iNKT to Tfh in the spleen on Day 28 decreased in the vehicle group significantly. We assumed it might reflect the aggressive increase of Tfh in the vehicle group which was consistent with the development of skin cGVHD. The relative proportion of Treg to Tfh in the spleen on Days 28 and 56 increased in the lipo α‐GC group significantly. We assumed it might reflect the increased Treg and the decreased Tfh in the lipo α‐GC group which was consistent with our hypothesis that increased Treg could suppress Tfh and skin cGVHD.

The relative proportion of CD4^+^ T cells, Treg cells, Tfh cells, and GCB cells to total lymphocyte on Days 7, 28, and 56 in the spleen (Figure S5) and in MLNs (Figure S6) are shown, respectively. The relative proportion of CD4^+^ T cells and Treg cells increased in the spleen on Day 56 in the lipo α‐GC group significantly. The relative proportion of Tfh on Days 28 and 56 and GCB on Day 56 in MLNs decreased in the lipo α‐GC group significantly. We assumed that these data were consistent with our hypothesis that lipo α‐GC may suppress the increase of Tfh and GCB and prevent the development of chronic GVHD. In previous studies, NKT‐cell‐depletion abolished the increase of Treg by α‐GC and the suppression of acute GVHD. We assume that NKT‐cell‐depletion could abolish the reduction of Tfh and GCB and the suppression of chronic GVHD as well, however it should be warranted by future studies.

The differential effect of lipo α‐GC on donor‐ and host‐type iNKT cells is considered to be complicated. Previous murine studies have suggested that stimulation of host‐type iNKT cells by α‐GC play an important role in preventing acute GVHD, but have not shown the kinetics of host‐ and donor‐type iNKT after the injection of α‐GC. In this study, we showed that lipo α‐GC treatment resulted in the reduction of host‐type iNKT and the increase of donor‐type iNKT in spleen and lymph nodes (Figures 3 and S8). As described above, we hypothesize that lipo α‐GC may promote the immigration of host‐type iNKT from the lymphoid organ to the GVHD target organ and on the other hand promote the increase of donor‐type iNKT in the lymphoid organs. Further research will be needed to warrant this consideration.

In general, the inflammatory state can hamper Treg homeostasis and reduced the population as shown in autoimmune murine models including OVA‐induced bronchial asthma and experimental autoimmune encephalomyelitis and colitis.^40^, ^41^, ^42^ Furthermore, Treg decrease can result in the increase of Tfh through the reduced function of CTLA‐4.^23^ As well as autoimmune diseases, altered inflammatory environment after allogeneic HSCT hamper Treg homeostasis.^43^ Our data from this study demonstrated that lipo α‐GC increased Treg and decreased Tfh irrespective of the intensive inflammation early after HSCT. We consider it is an important finding for the clinical appliance of lipo α‐GC.

In conclusion, our data showed that multiple lipo α‐GC administrations altered the early homeostasis of both host‐ and donor‐derived iNKT cells and effectively prevented sclerodermatous cGVHD through donor Treg expansion, followed by the inhibition of Tfh and GCB cells. This study demonstrated an important coordination of T‐ and B‐cell immunity in the pathogenesis of cGVHD and may provide a novel clinical strategy for the induction of immune tolerance after allogeneic HSCT.

## CONFLICT OF INTERESTS

Yasuyuki Ishii is the CEO of REGiMMUNE. The remaining authors declare that there are no conflicts of interests. 

## ETHICS STATEMENT

All animal experiments were performed according to the regulations of the Animal Care and Use Committee, Okayama University Advanced Science Research Center.

## AUTHOR CONTRIBUTIONS

Hiroyuki Sugiura designed and performed the main experiments, and wrote the paper. Takuya Fukumi designed and led the additional experiments. Yuichi Sumii, Takumi Kondo, and Shuntaro Ikegawa performed the additional experiments and edited the paper. Yusuke Meguri, Miki Iwamoto, Yasuhisa Sando, and Makoto Nakamura helped experiments. Tomohiro Toji scored skin pathological score. Yasuyuki Ishii provided lipo α‐GC for the study and supervised the laboratory studies; Yoshinobu Maeda supervised the laboratory studies and edited the paper; Ken‐ichi Matsuoka designed and supervised the research project and edited the paper.

## Supporting information

Supporting information.

## Data Availability

The datasets during the current study available from the corresponding author on reasonable request.
