# Supplementary material for: Donor Treg expansion by liposomal α‐galactosylceramide modulates Tfh cells and prevents sclerodermatous chronic graft‐versus‐host disease
Source: Immun Inflamm Dis. 2021 May 4;9(3):721–33. doi: 10.1002/iid3.425 (PMC8342231; doi:10.1002/iid3.425)
Supplement: Supplementary file 1 — Supporting information. [file IID3-9-721-s001.pdf]

Supplementary information for

Donor Treg expansion by liposomal α-galactosylceramide modulates T fh cells and prevents sclerodermatous chronic graft-versus-host disease

Hiroyuki Sugiura, Ken-ichi Matsuoka, Takuya Fukumi, Yuichi Sumii, Takumi Kondo, Shuntaro Ikegawa, Yusuke Meguri, Miki Iwamoto, Yasuhisa Sando, Makoto Nakamura, Tomohiro Toji, Yasuyuki Ishii and Yoshinobu Maeda

This file includes:

Supplemental figures 1-8

Corresponding Author:

Ken-ichi Matsuoka M.D., Ph.D

Department of Hematology and Oncology, Okayama University Graduate School of Medicine, Dentistry, and Pharmaceutical Sciences, Shikata-cho 2-5-1, Kita-ku, Okayama-city, Okayama, Japan

Phone :+81-86-235-7227   Fax:+81-86-232-8226

E-mail: k-matsu@md.okayama-u.ac.jp

Supplemental figure1.

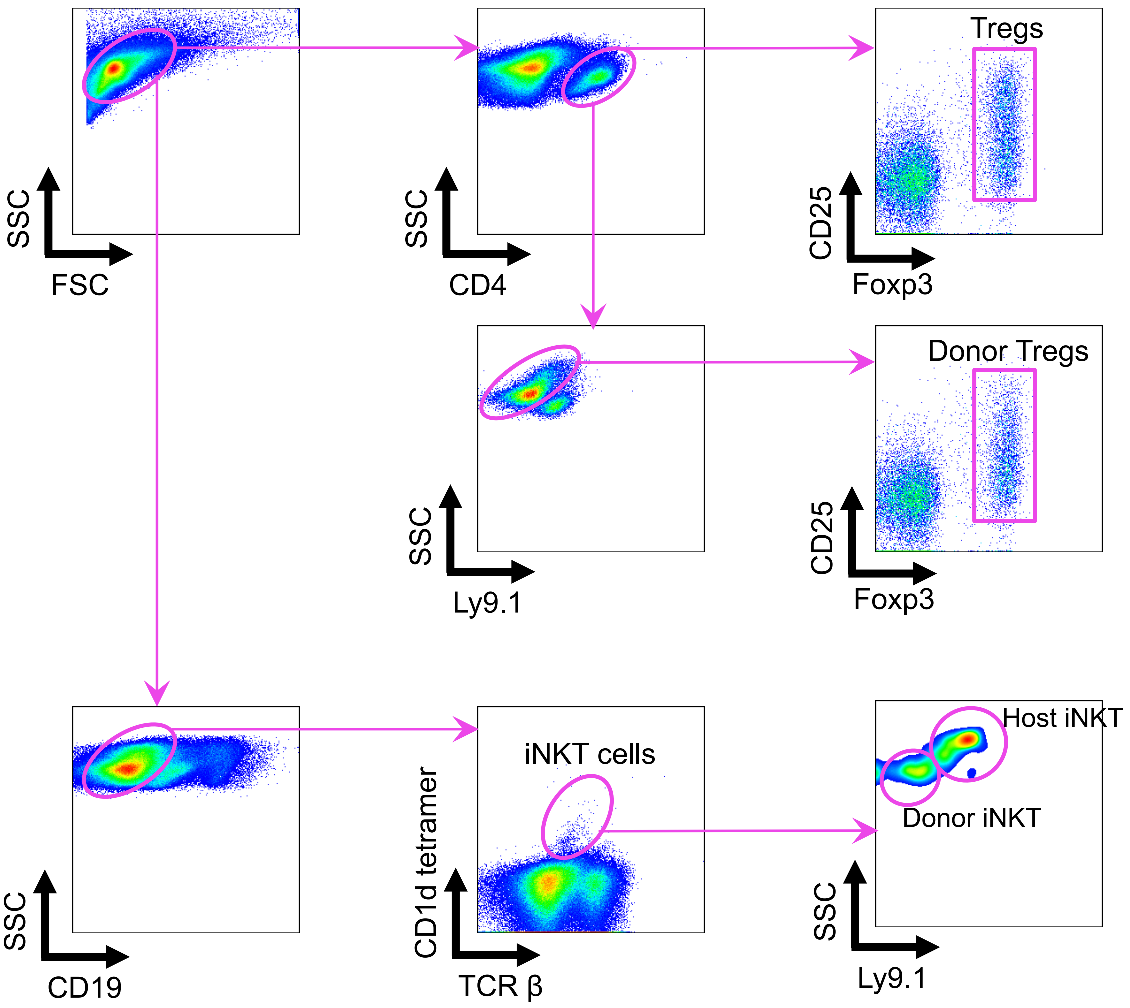

Gating strategy for Tregs and iNKT cells.

Tregs are indentified as $CD4^{+}CD25^{med\text{-}high}Foxp3^{+}$. Donor-derived $CD4^{+}$ T cells and Tregs are identified by the expression pattern of Ly9.1.

iNKT cells are indentified as $CD19^{-}TCR\beta^{-}CD1d\text{-}tetramer^{+}$ and further divided into donor-derived and host-derive subsets by the expression pattern of Ly9.1.

Supplemental figure2.

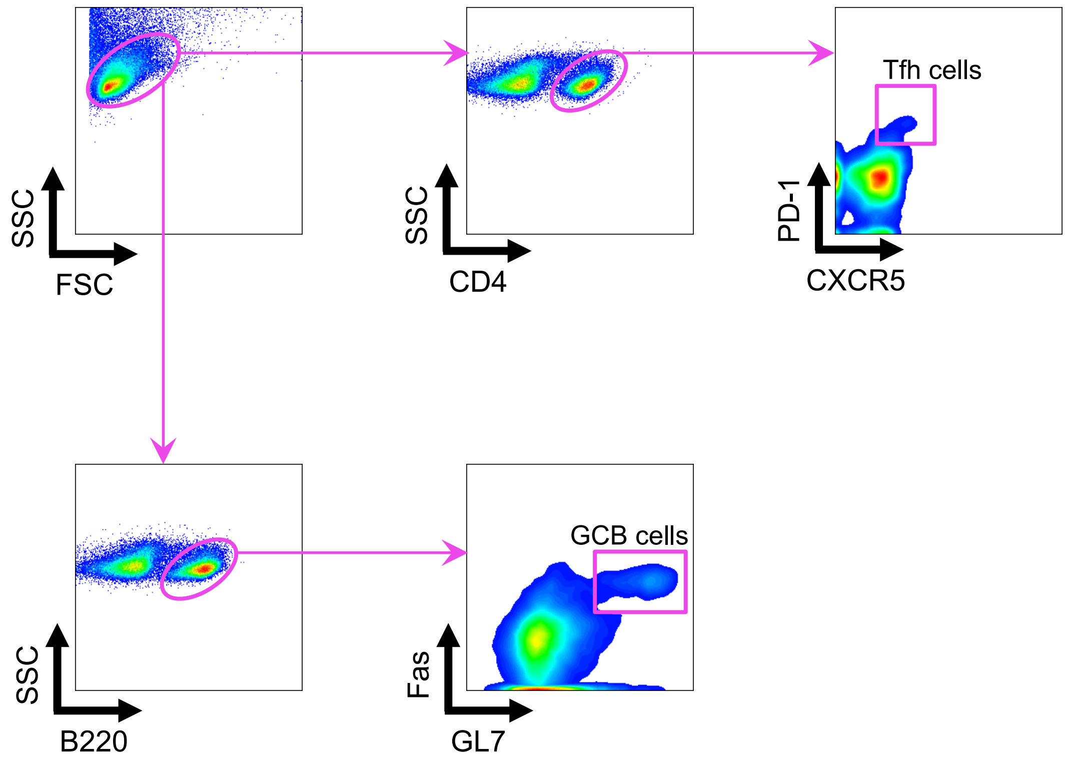

Gating strategy for Tfh cells and GCB cells.

T follicular helper cells (Tfh cells) are indentified as $CD4^{+}CXCR5^{med\text{-}high}PD\text{-}1^{+}$. GCB cells are indentified as $B220^{+}GL7^{+}Fas^{+}$.

Supplemental figure 3.

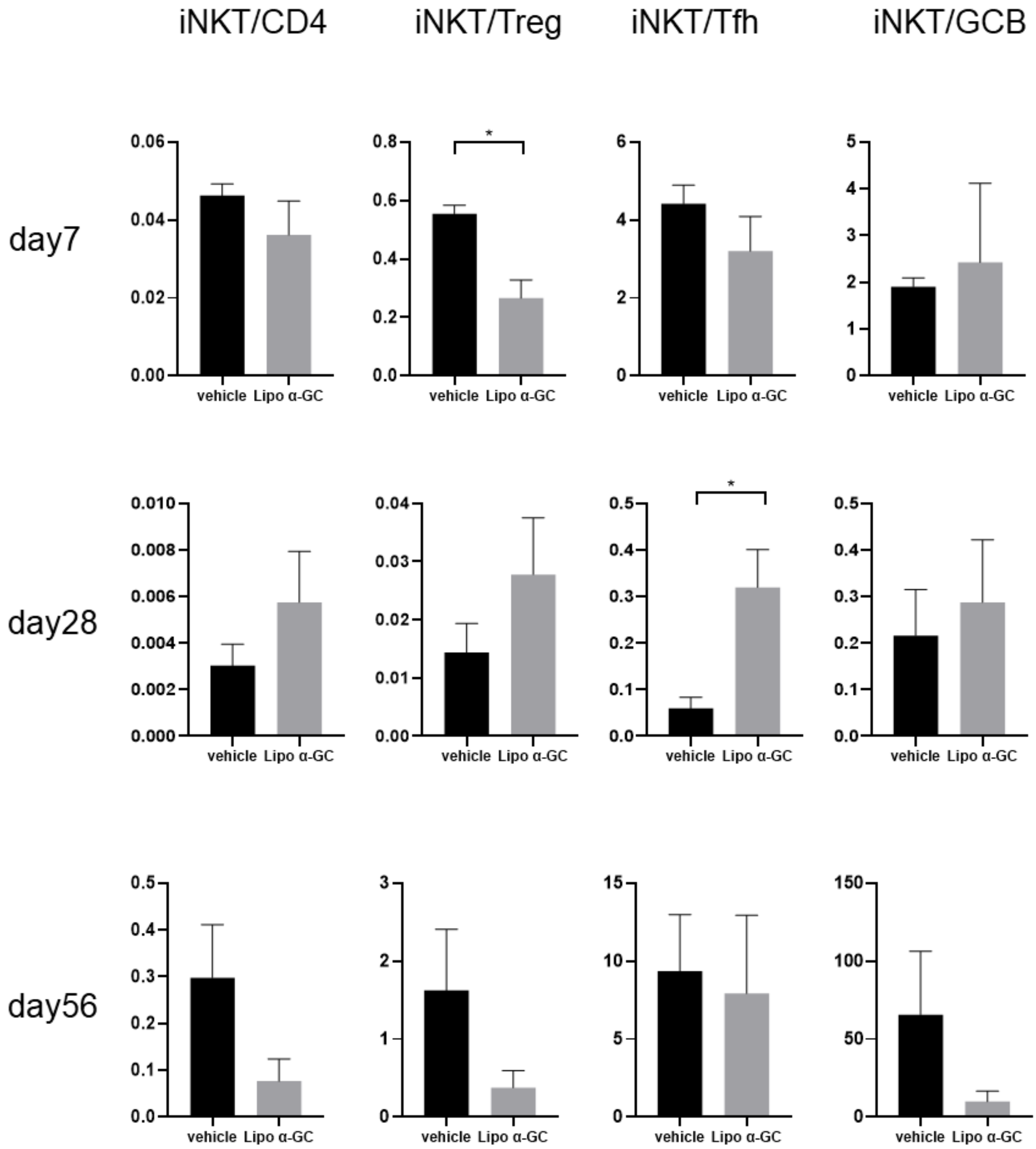

The relative proportion of iNKT to CD4, Treg, Tfh and GCB in spleen on day7, 28, and 56.

The relative proportion of iNKT to Treg in spleen on day7 decreased in lipo α-GC group significantly, and the relative proportion of iNKT to Tfh in spleen on day28 decreased in vehicle group significantly. Statistical analysis was performed by Student's t-test.*P<0.05

Supplemental figure 4.

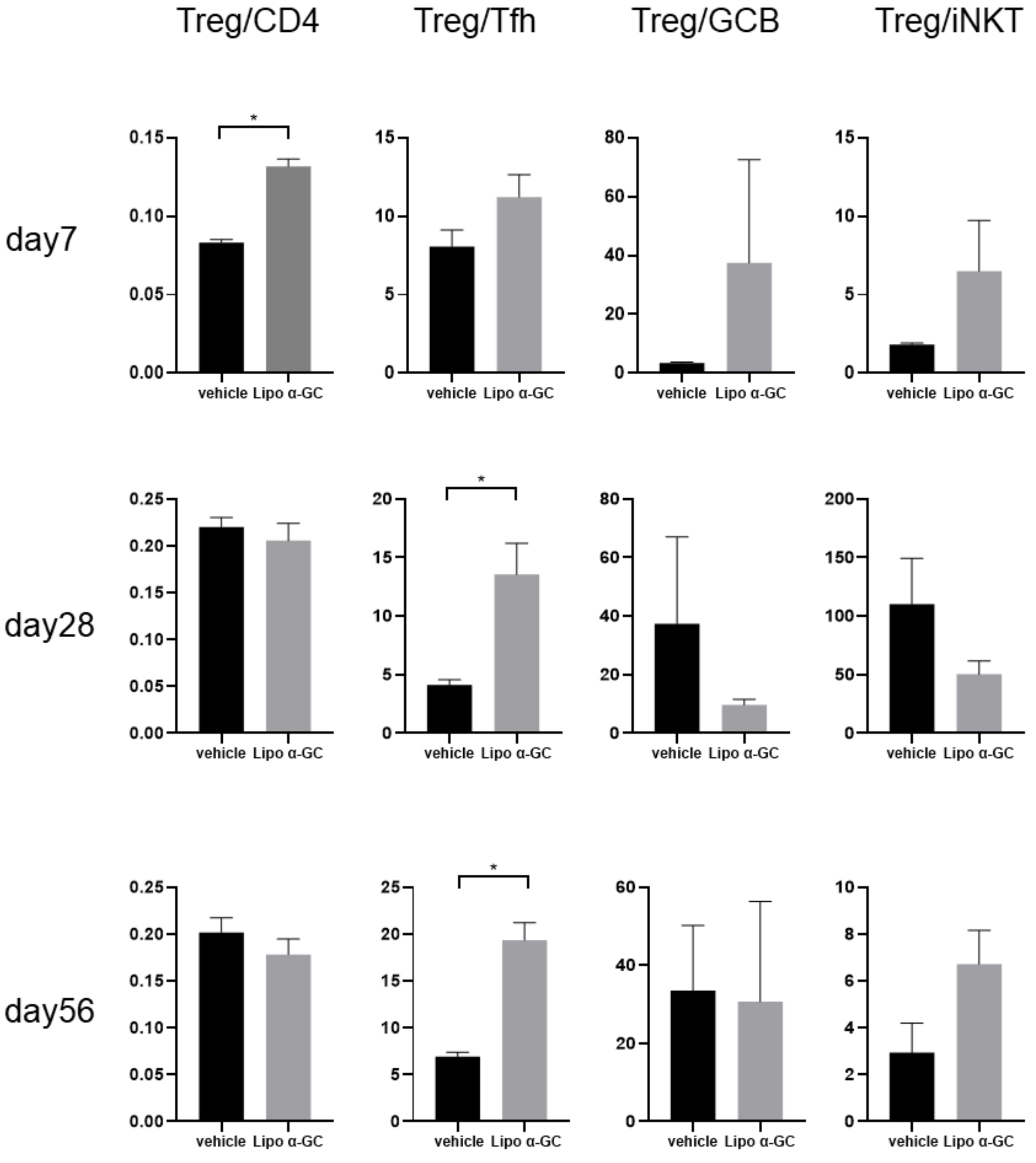

The relative proportion of Treg to CD4, Tfh, GCB and iNKT in spleen on day7, 28, and 56.

The relative proportion of Treg to CD4 increased in lipo α-GC group significantly on day7 and the relative proportion of Treg to Tfh in spleen on day28, 56 increased in lipo α-GC group significantly. Statistical analysis was performed by Student's t-test.*P<0.05

Supplemental figure 5.

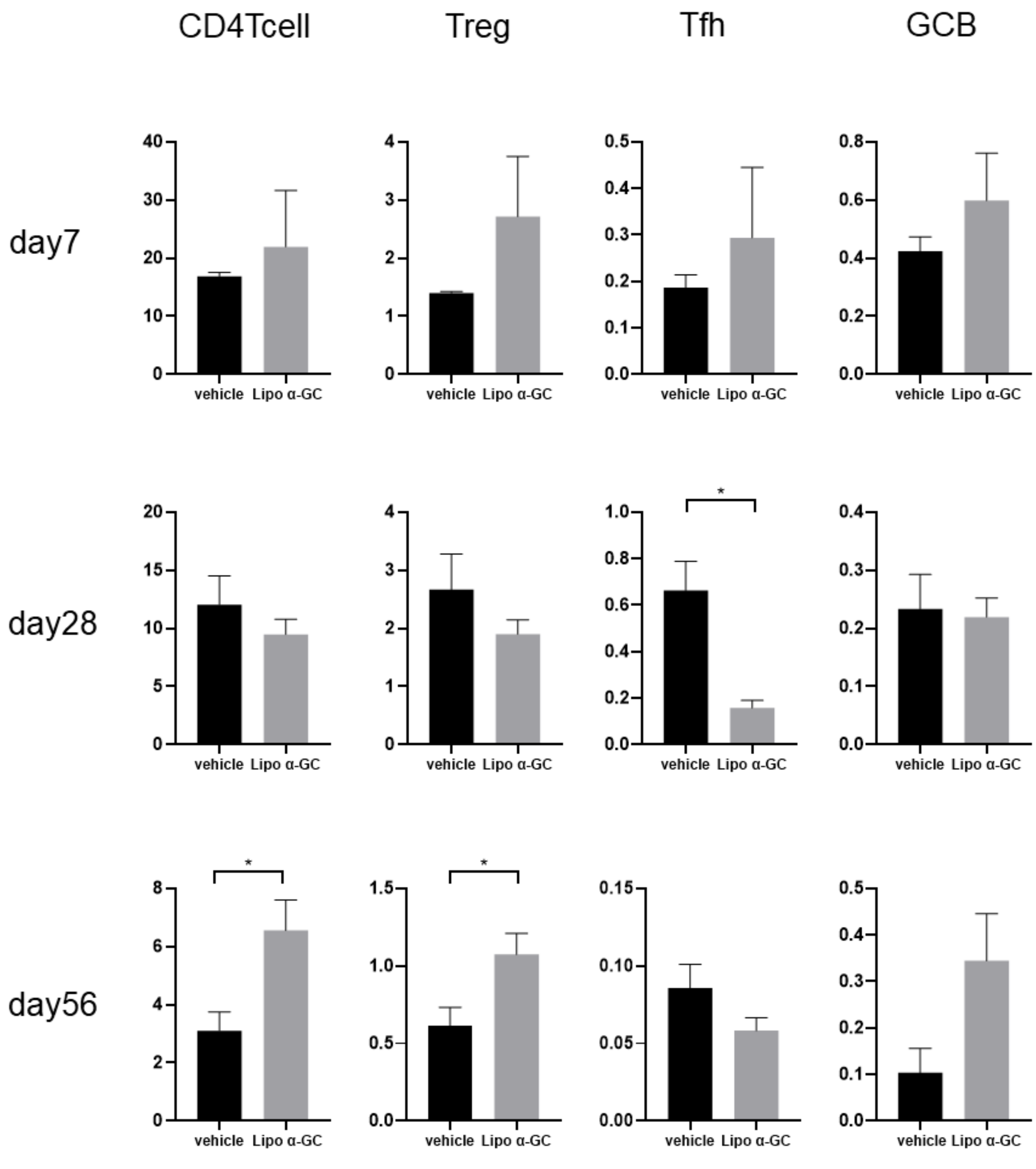

The relative proportion of CD4, Treg, Tfh and GCB to total lymphocyte on day7, 28, and 56 in spleen.

The relative proportion of CD4 and Treg increased in spleen on day56 in lipo α-GC group significantly and the relative proportion of Tfh on day28 decreased in lipo α-GC group significantly. Statistical analysis was performed by Student’s t-test.*$P<0.05$

Supplemental figure 6.

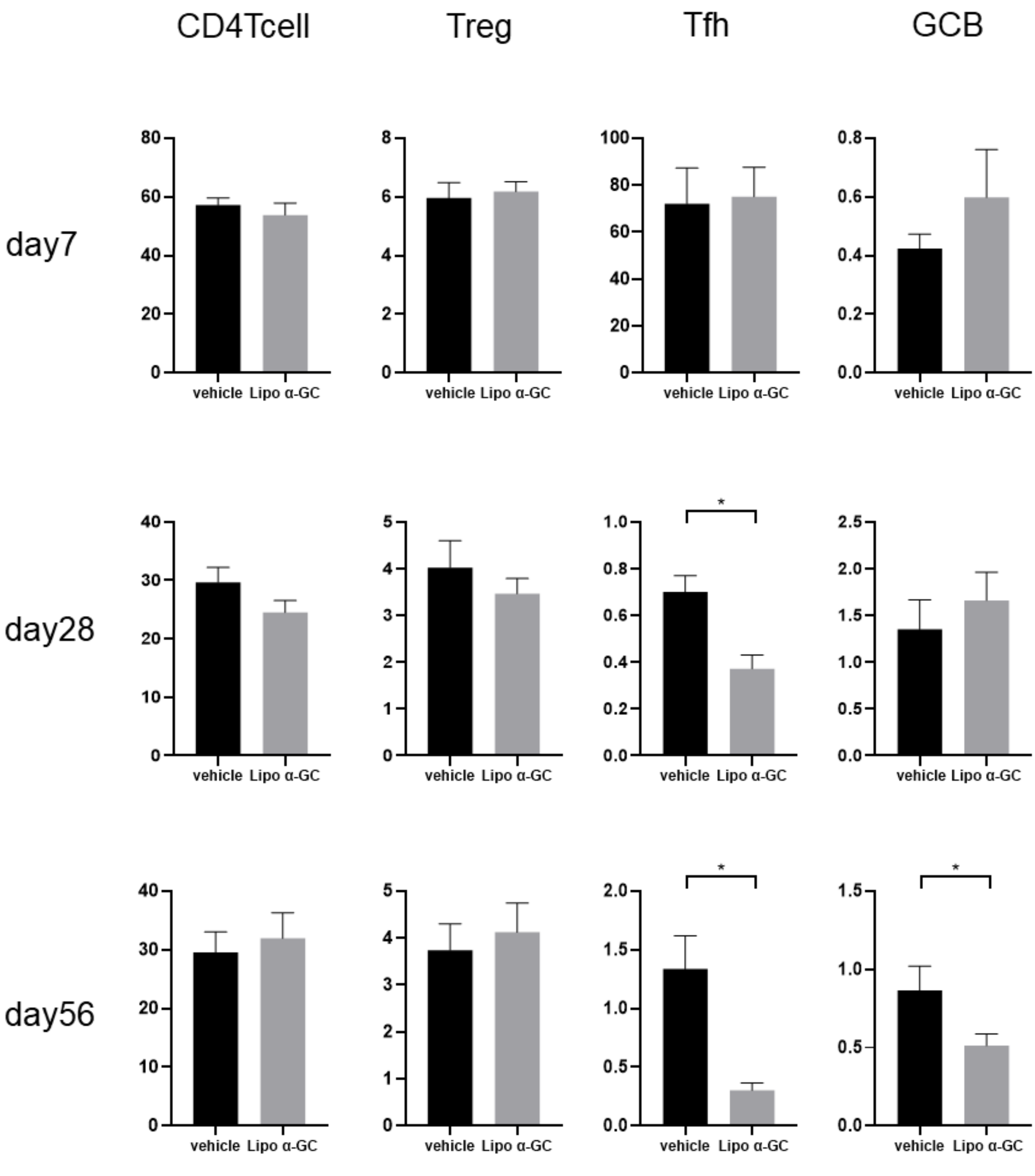

The relative proportion of CD4, Treg, Tfh and GCB to total lymphocyte on day7, 28, and 56 in MLN.

The relative proportion of Tfh on day28 ,56 and GCB on day56 in MLNs decreased in lipo α-GC group significantly. Statistical analysis was performed by Student's t-test.*P<0.05

Supplemental Figure 7.

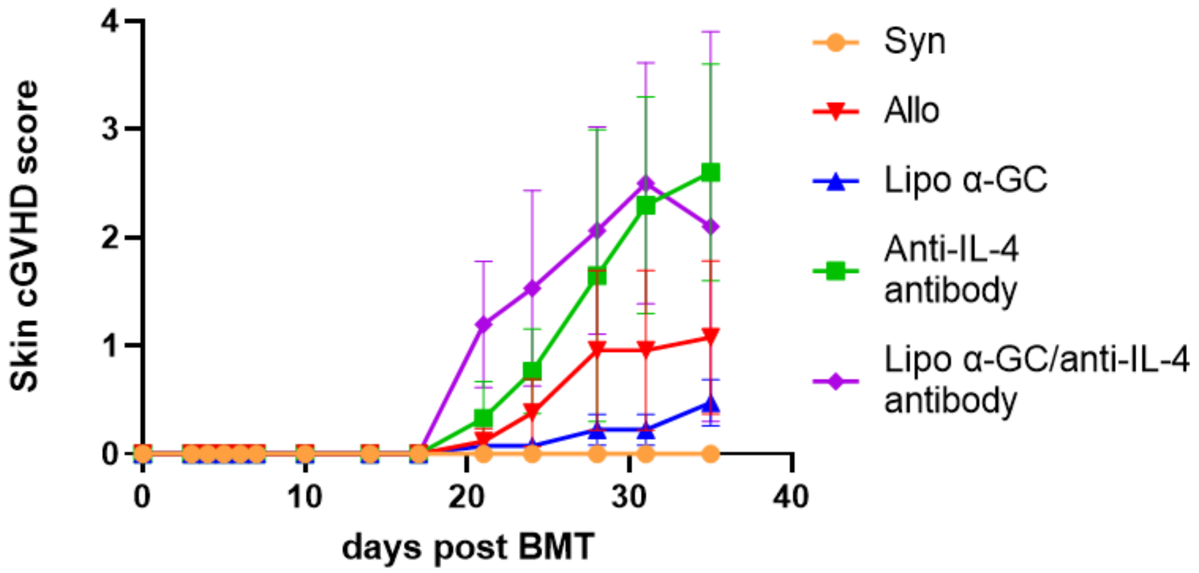

Preventive effect of lipo α-GC against cGVHD was cancelled by anti-IL-4 antibody. BALB/c recipient mice received 6 Gy of TBI; then, 8×10^6 BM cells and 10×10^6 spleen cells were administered from syngeneic Balb/c mice or allogeneic B10.D2 donor mice. Lipo α-GC was administered at a dose of 1 μg/kg via tail vein injection on days 0, 3, and 5 after BMT. Anti-IL4 antibody was administered intraperitoneally on day 0 (2mg/body), and day 3 and 5 (1mg/body) after BMT. Time courses of skin cGVHD score after BMT in syngeneic (n = 3), allogeneic (n = 10), allogeneic/lipo α-GC (n = 10), allogeneic/anti-IL-4 antibody (n = 6), and allogeneic/lipo α-GC/anti-IL-4 antibody (n = 6) groups. Data are expressed as the means ± SEM.

Supplemental Figure 8.

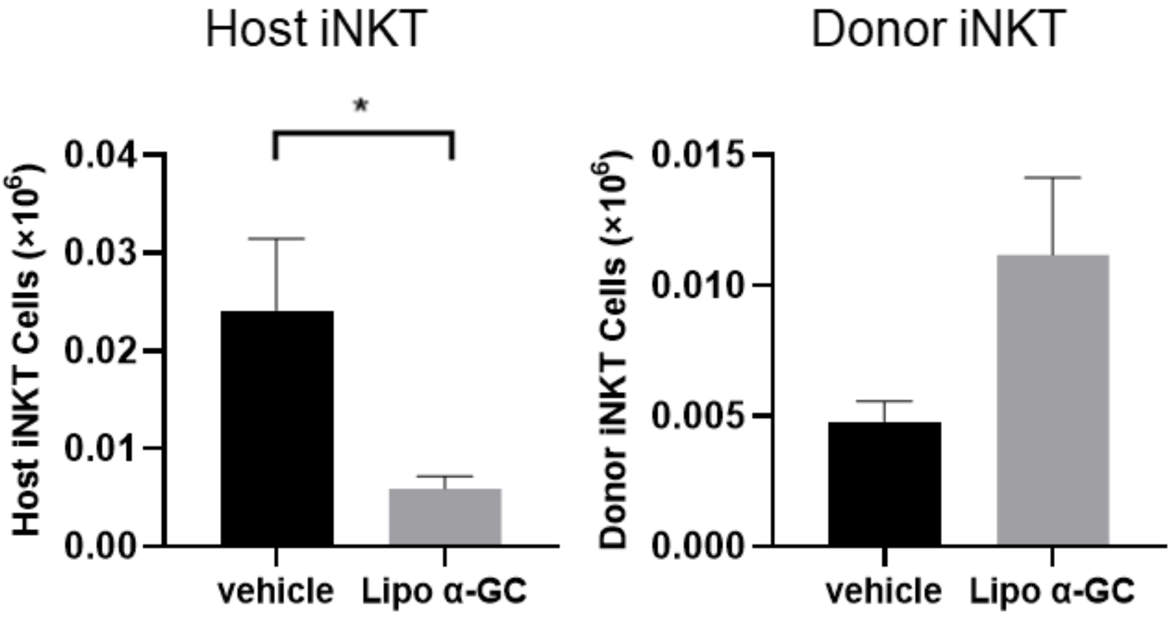

Lipo α-GC increases donor iNKT cells and decreases host iNKT cells in lymph nodes in an early phase after BMT.

INKT cells were defined as CD19−TCR-β+CD1d tetramer+ cells. Chimerism was evaluated in the host (Ly9.1+) or donor (Ly9.1−). The number of donor iNKT cells and host iNKT cells in MLNs after BMT in vehicle (n = 4) and lipo α-GC (n = 4) mice on day 7. Data are expressed as the means ± SEM. P-values were determined using an unpaired, two-tailed Student's t-test. *$P < 0.05$.
